# Movement Control Strategies of the Mawashi Geri Jodan in Female Karate Athletes

**DOI:** 10.3390/sports14040134

**Published:** 2026-03-31

**Authors:** Linguo Chen, Hongwei Yan, Yuqiao Zhu, Wei Shan

**Affiliations:** 1 Sport Coaching College, Beijing Sport University, Beijing 100048, China; chenlinguo777@163.com (L.C.); yanhongwei@bsu.edu.cn (H.Y.); felix17202411@163.com (Y.Z.); 2 Laboratory of Sports Stress and Adaptation of General Administration of Sport, Beijing Sport University, Beijing 100084, China; 3 China Institute of Sport and Health Science, Beijing Sport University, Beijing 100084, China

**Keywords:** female karate athletes, Mawashi Geri Jodan, kinesiology, surface electromyography

## Abstract

Among lower-body techniques in karate, the Mawashi Geri Jodan is regarded as the most frequently applied, technically sophisticated, and potentially hazardous skill. Yet, whether karate athletes of varying proficiency levels exhibit differential mastery of this technique remains empirically unexamined. This study aimed to reveal movement control strategies of elite athletes by comparing kinematic and surface electromyography (sEMG) characteristics of Mawashi Geri Jodan between elite and sub-elite female karate practitioners. A total of eight female karate athletes (4 elite, 4 sub-elite) were recruited. During the execution of the dominant-leg Mawashi Geri Jodan, they struck a karate punching bag positioned at head height, while kinematic and sEMG data were synchronously collected. Analyzed metrics included phase durations, center of mass (COM) displacement, joint angles/angular velocities, and integral electromyography (IEMG) with muscle work percentage of 8 lower limb muscles. Independent-sample *t*-tests were used for intergroup comparisons (α = 0.05). Compared with the sub-elite group, elite athletes completed the full Mawashi Geri Jodan in significantly less time (0.825 ± 0.07 s vs. 1.030 ± 0.05 s, *p* < 0.01) and exhibited a shorter core striking phase (*p* < 0.05). Kinematically, elite athletes showed smaller vertical COM displacement during the striking phase (*p* < 0.05) and greater hip joint range of motion (*p* < 0.05). sEMG data revealed significantly higher activation of lower limb prime movers (vastus lateralis, gastrocnemius) during the striking phase and greater rectus femoris contribution during the recovery phase in elite athletes. Elite female karate practitioners demonstrate superior movement efficiency, body stability, and neuromuscular coordination in Mawashi Geri Jodan. Technical training should prioritize hip joint flexibility and stability, synergistic explosive force generation of the lower limb kinetic chain during the striking phase, and active rectus femoris activation during the recovery phase to enhance execution precision and efficiency.

## 1. Introduction

Karate is a martial art focused on kumite (sparring) and kata (form) training, requiring rapid reactions, synchronized attack–defense coordination, and sophisticated motor skills (Molinaro et al., 2020) [1]. Karate kumite involves competitive bouts between two athletes under standardized rules, with points awarded for high-speed, powerful technical actions. Full-force strikes to the torso are permitted, while controlled contact is mandated for head strikes (Spigolon et al., 2018) [2]. Characterized by high intensity, kumite movements are explosive and intermittent, with individual actions lasting 0.3–2.1 s, demanding exceptional technical precision and tactical application (Ojeda Aravena et al., 2019) [3].

The World Karate Federation (WKF) scoring system categorizes points as 1 point (yuko), 2 points (waza-ari), and 3 points (ippon), with the highest “ippon” typically awarded for effective leg techniques targeting the head [4]. Like most combat sports, karate competitions integrate technique, power, and speed (Beekley et al., 2006) [5]. Lower-body explosive power is a key determinant of success, directly influencing kick velocity and force, thus becoming a primary prerequisite for scoring (Loturco et al., 2014) [6]. Elite karate athletes often prioritize lower-body techniques (e.g., head and torso kicks) to secure higher scores in kumite, gaining a significant strategic advantage (Tabben et al., 2014; Błaszczyszyn et al., 2019) [7,8]. Consequently, karate competitions require athletes to possess high levels of explosive power, technical precision, and tactical execution (Koropanovski et al., 2011) [9].

The Karate Physical Fitness Test (SPFT) includes a flexibility assessment for Mawashi Geri (Story G, 1989) [10]. As a critical technique for striking the head, face, and neck, the Mawashi Geri’s effectiveness is particularly notable. Among lower-body techniques, the Mawashi Geri Jodan is regarded as the most frequently used, effective, and potentially dangerous (Hariri & Sadeghi, 2018) [11]. However, relevant data indicate that while this technique accounts for 21.95% of leg techniques employed in competitions, it contributes to only 2.34% of scoring points (Zhou, 2020) [12]. This highlights that the operational efficiency of the Mawashi Geri among high-level athletes is a pressing practical issue requiring resolution. The effectiveness of the Mawashi Geri largely depends on kinematic characteristics such as execution speed, joint angles, and body stability, which collectively determine striking power while reducing opponents’ reaction time (Provenza, 2020) [13].

Highly efficient punching and kicking techniques rely on well-developed muscular strength as a foundation (Martínez de Quel et al., 2020) [14]. Biomechanical research provides objective analytical tools to thoroughly analyze and optimize techniques. For instance, kick duration can serve as an indicator for identifying elite athletes (Pozo et al., 2011) [15]. Kinematic analysis quantifies key parameters, including hip and knee angular velocities, center-of-mass (COM) displacement, and phase durations. Meanwhile, surface electromyography (sEMG) reveals activation patterns of agonist and stabilizer muscles during movement (Kim et al., 2010) [16]. Previous studies have advocated for the integration of 3D video analysis into training for motion correction (Sforza et al., 2002) [17], and sEMG data analysis offers valuable insights for optimizing training strategies (Sbriccoli et al., 2010) [18]. Synchronous analysis of kinematics and sEMG has proven effective in deconstructing the biomechanical characteristics of karate techniques, such as punch movements (VencesBrito et al., 2011) [19].

Most current research in combat sports has predominantly focused on male athletes (Corcoran et al., 2024) [20]. Due to inherent sex-based differences in physiological characteristics and movement strategies, findings derived from male populations cannot be directly generalized to female athletes (MacGregor et al., 2025) [21]. In karate, it has been observed that high-intensity actions, defined as rapid and forceful offensive and defensive techniques, are performed at a higher magnitude by male compared to female athletes (Tabben et al., 2015) [22]. Furthermore, distinct neuromuscular coordination and motor control patterns exist between sexes (Ferreira & Vencesbrito, 2012) [23]. Although a substantial body of biomechanical research has been conducted on male karate athletes (Robalino et al., 2025; Vuković et al., 2021; Diniz et al., 2021; Zago et al., 2015) [24,25,26,27], studies focusing on female practitioners, particularly those examining the physical and biomechanical profiles of elite-level athletes, remain critically insufficient. This scarcity underscores the necessity of investigating the specific characteristics of female kumite competitors (Chaabène et al., 2012) [28].

There are significant differences in biomechanical indicators among athletes of different skill levels. Research shows that the smaller the center-of-mass displacement, the better the postural stability (Zago et al., 2014) [29]. Hip joint range of motion was found to be highly correlated with expert technical scores (*p* > 0.8) and could effectively distinguish between skill levels (Hölbling et al., 2023) [30]. Elite taekwondo athletes exhibit higher levels of muscle activation during roundhouse kicks (ES = 0.53–0.66) (Sun et al., 2025) [31], and the activation patterns of key muscle groups are also associated with kicking performance (Zhang et al., 2025) [32].

In sports science research, the classification of “elite” and “sub-elite” athletes is commonly established based on competitive achievement and training background. Elite athletes are generally defined as those who have attained top-level performance standards, evidenced by medals or placements in international or national competitions, combined with years of systematic training. Sub-elite athletes, while possessing considerable technical proficiency, have not yet reached the competitive pedigree or training volume characteristic of their elite counterparts (Swann et al., 2015) [33]. Within the context of karate, previous studies adopting similar classification criteria have reported significant differences between elite and sub-elite practitioners in kinematic and electromyographic parameters during high-intensity technique execution (VencesBrito et al., 2011; Quinzi et al., 2014) [19,34].

The specific aims of this study were to reveal differences in key kinematic parameters and sEMG characteristics during Mawashi Geri Jodan execution among female karate practitioners of varying skill levels through comparative analysis.

## 2. Materials and Methods

### 2.1. Study Design

A cross-sectional comparative design was employed to examine differences in kinematic and surface electromyographic characteristics during Mawashi Geri Jodan execution between elite and sub-elite female karate athletes. Participants were categorized into elite (*n* = 4) and sub-elite (*n* = 4) groups based on competitive achievement and training background. All athletes completed the testing protocol during a single session.

### 2.2. Participants

The minimum sample size was calculated using G*Power 3.1.9.4 software (University of Düsseldorf, Düsseldorf, Germany) for an independent-sample *t*-test with the following parameters: α = 0.05, effect size = 2.00, power (1 − β) = 0.80. This analysis indicated a minimum sample size of 6 participants to achieve statistical significance. A total of eight female karate athletes were recruited (Table 1). The sample size (N = 8) is consistent with previous biomechanical studies on karate techniques (Portela et al., 2014, N = 8; Valdés-Cabrera et al., 2020, N = 6) [35,36]. Given the limited pool of elite athletes, small samples are inherent in such research; rigorous design and effect size reporting ensure meaningful findings (Skorski et al., 2021; Hecksteden et al., 2022) [37,38]. Based on the Chinese National Athlete Technical Classification System (General Administration of Sport of China, 2024) [39] and established definitions of elite and sub-elite athletes (Swann et al., 2015) [33], participants were divided into two groups:

Elite Group (E): 4 “National-Class Athletes” (top-tier in Chinese karate, with outstanding performances in national championships and games).

Sub-elite Group (F): 4 “National Class I Athletes” (consistently outstanding performers in provincial-level competitions).

Inclusion criteria required participants to be active athletes with at least one year of systematic strength and karate-specific training (≥3 sessions per week), participation in the most recent national championship, confirmation of no lower limb injuries in the past six months through standardized musculoskeletal screening (American College of Sports Medicine, 2021) [40], and no significant weight loss (>10% body mass) during the same period. All participants completed the PAR-Q questionnaire to rule out vigorous exercise, smoking, alcohol consumption, or caffeine intake within 48 h prior to data collection.

All participants were fully informed of the study procedures, risks, and benefits and provided written informed consent. All protocols adhered to the Declaration of Helsinki and were approved by the Ethics Committee for Sports Science Experiments of Beijing Sport University (Approval Number: 2025106H).

### 2.3. Experimental Procedure

This cross-sectional study was conducted in a standardized karate training venue during the mid-season, with all tests performed between 9:00 and 12:00 to control for circadian rhythm effects. The experimental environment temperature was maintained at 24–26 °C to mitigate temperature-related impacts on muscular performance and skin impedance.

Anthropometric data were collected using an HC Height and Weight Measuring Device (Model: HCM-901, China, Shanghai Huachao Electric Co., Ltd., Shanghai, China) prior to the testing warm-up. Height and body mass were measured by the same researcher, following the guidelines of the International Society for the Advancement of Kinanthropometry (Stewart et al., 2011) [41]. Prior to testing, all participants completed a 15 min warm-up based on the RAMP principle (Jeffreys, 2007) [42], including low-intensity cardio (jumping jacks, brisk walking), dynamic stretching (leg swings, hip circles, knee extensions), and submaximal Mawashi Geri Jodan practice (Hibbs et al., 2008) [43]. Participants then wore a sports vest and black compression leggings for data collection.

#### 2.3.1. sEMG Data Collection

**Figure 1:**
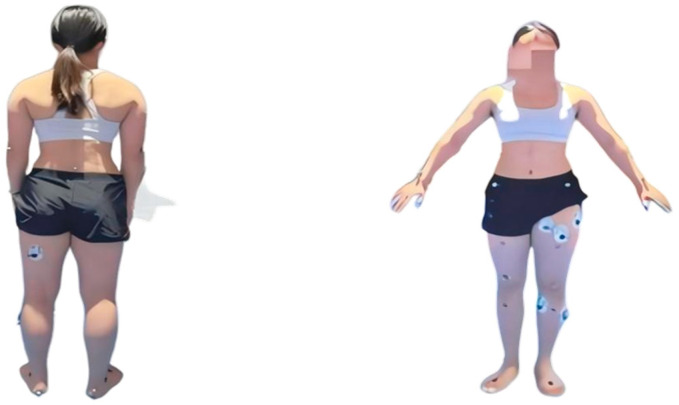
The pre-test preparation status of the experimental participants.

A wireless sEMG system (Zhiyunwei, YW-Wireless, China) was used to synchronously acquire electromyographic signals at a sampling frequency of 1000 Hz. Electrode placement strictly followed SENIAM project guidelines (Hermens et al., 2000) [44]: skin sites were disinfected with alcohol swabs, exfoliated with emery paper, and re-cleaned with alcohol to reduce impedance (Mao et al., 2017) [45]. After natural drying, bipolar electrode patches (2 cm apart) were affixed parallel to muscle fiber orientation at the mid-belly of 8 target muscles of the attacking leg: gluteus maximus, rectus femoris, vastus lateralis, vastus medialis, biceps femoris, tensor fasciae latae, gastrocnemius, and sartorius (Hariri & Sadeghi, 2018; Han, 2023) [11,46]. As shown in Figure 1, anatomical localization referenced relevant studies and anatomical atlases.

#### 2.3.2. Three-Dimensional Motion Capture

An 8-camera 3D motion capture system (NOKOV, China, Beijing NOKOV Science & Technology Co., Ltd., Beijing, China) with a sampling frequency of 100 Hz was employed (Zhang & Guan, 2022; Ting et al., 2023) [47,48]. Participants stood in a standard anatomical posture at the center of the testing area for static acquisition, defining local coordinate systems and joint centers for each segment. The 39-point full-body marker set from the Plug-in Gait model was used (Zhou et al., 2024) [49], with additional tracking markers placed on both iliac crests, iliac spines, and lateral aspects of the thighs and calves to enhance the capture of pelvic and lower limb rotation during kicking, as shown in Figure 1.

#### 2.3.3. Kick Execution

A standard karate punching bag was used as the target, with height adjusted to the participant’s head height and the initial horizontal distance set to allow striking with one forward step. Participants initiated the experiment in a standardized left-side standing posture and executed Mawashi Geri Jodan with their dominant (left) leg upon hearing the “start” command. Each participant performed 5 valid kicks, with a 60 s rest period between attempts to ensure phosphagen system recovery and prevent fatigue (Falco et al., 2009) [50]. All kicks were supervised by a national-level referee and met validity criteria: (1) terminal kicking velocity > 10–15 m/s; (2) technical execution conforming to competition standards.

### 2.4. Technical Phase Classification

**Figure 2:**
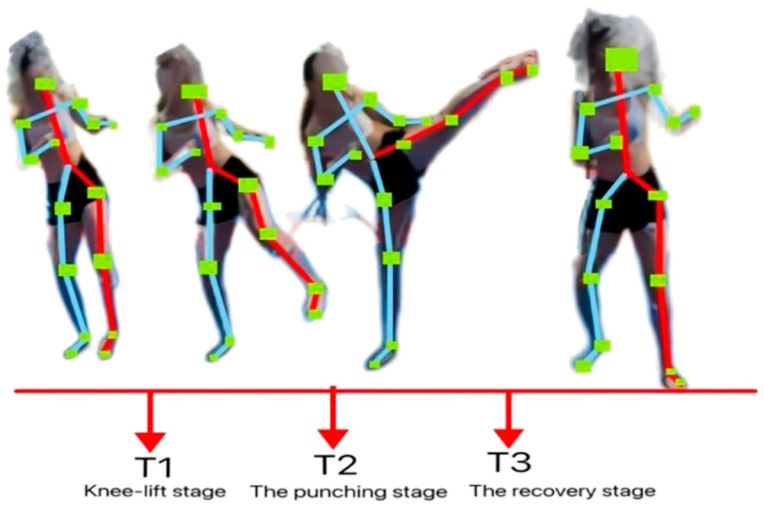
The diagram of the experimental setup illustrates the initial position of the subject, facing the beanbag and preparing to perform Mawashi Geri Jodan, and the final position of the motion, in which the leg makes contact with the target.

Based on karate movement requirements and Mawashi Geri Jodan characteristics (Valdés 2020) [36], combined with expert interviews and combat application scenarios (counterattack-focused), the technique was divided into three phases, as shown in Figure 2:

Knee Lift Phase: From the initiation of knee lift to the start of striking.

Striking Phase: From the start of striking to the completion of target contact.

Recovery Phase: From the completion of striking to the full return to the initial stance.

### 2.5. Data Analysis

To ensure reliability, all 5 valid kicks per participant were analyzed. Test–retest intraclass correlation coefficients ranged from 0.85 to 0.94 for kinematic variables and 0.82 to 0.91 for sEMG, indicating good reliability. Standard measurement errors were 2.1–3.8° for joint angles and 2.5–4.8 μV·s for integrated EMG, confirming acceptable precision for between-group comparisons. All 5 valid kick datasets were included for analysis. Raw kinematic data were processed (marker labeling, patching, filtering) using XINGYING 3.2 software (NOKOV) with a fourth-order low-pass Butterworth filter (cutoff frequency: 10 Hz). The motion capture system is calibrated in accordance with the manufacturer’s specifications prior to each data acquisition session, with a calibration error of less than 0.5 mm, and exported to Excel. Analyzed kinematic metrics included phase durations, COM displacement, joint angles, and peak joint angular velocities.

For surface electromyography signals, all sensor placements followed the SENIAM guidelines and were performed by the same experienced researcher to minimize inter-trial variability. The signal-to-noise ratio of the surface electromyography system was verified prior to each test. sEMG signals underwent bandpass filtering (20–450 Hz), full-wave rectification, and linear envelope processing (low-pass filter: 6 Hz) to obtain muscle activation levels. EMG data were normalized against the peak amplitude during maximal voluntary isometric contraction for each muscle. The analyzed sEMG metrics included IEMG and muscle work percentage.

All statistical analyses were performed using SPSS 25.0 software (IBM, Armonk, NY, USA). Data normality was verified via the Shapiro–Wilk test. Independent-sample *t*-tests were used to compare kinematic and sEMG parameters between groups, with Cohen’s d calculated to assess effect size (small: 0.2–0.5, medium: 0.5–0.8, large: >0.8). Statistical significance was set at α = 0.05. Data are presented as mean ± SD.

## 3. Results

Independent-sample *t*-tests were used to investigate differences in kinematic and sEMG parameters during Mawashi Geri Jodan execution between elite and sub-elite female karate practitioners. Despite the small sample size, participants represented a continuum of competitive levels (provincial to national elite), providing valuable insights into technical differences across high-performance tiers. Key findings with medium-to-large effect sizes are prioritized below.

### 3.1. Technical Action Times

**Figure 3:**
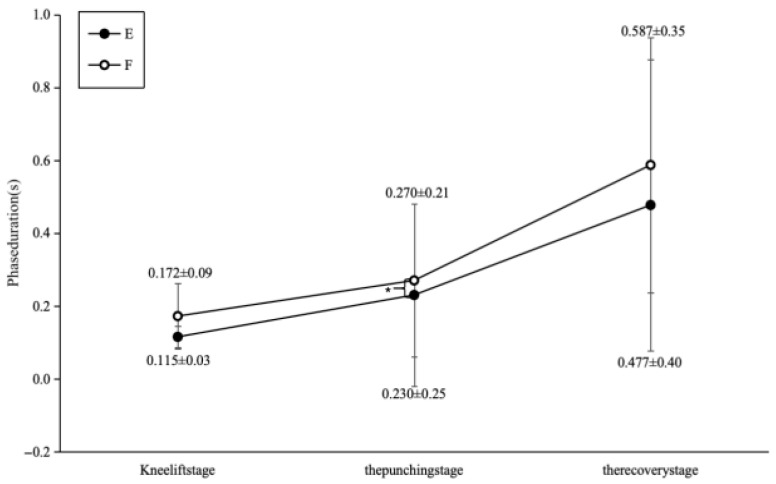
Comparison chart of technical action times between Group E and Group F.

The elite group completed the full Mawashi Geri Jodan in significantly less time than the sub-elite group (E: 0.825 ± 0.07 s vs. F: 1.030 ± 0.05 s; t(6) = 3.21, *p* = 0.005, Cohen’s d = 1.38; Figure 3). Elite athletes also exhibited shorter durations in the knee lift phase (0.115 ± 0.03 s vs. 0.172 ± 0.09 s), striking phase (0.230 ± 0.025 s vs. 0.270 ± 0.021 s), and recovery phase (0.477 ± 0.040 s vs. 0.587 ± 0.035 s) compared to the sub-elite group. Notably, the elite group showed a significantly shorter core striking phase (t(6) = 2.89, *p* = 0.01, Cohen’s d = 1.26).

### 3.2. Centre of Gravity Displacement

**Figure 4:**
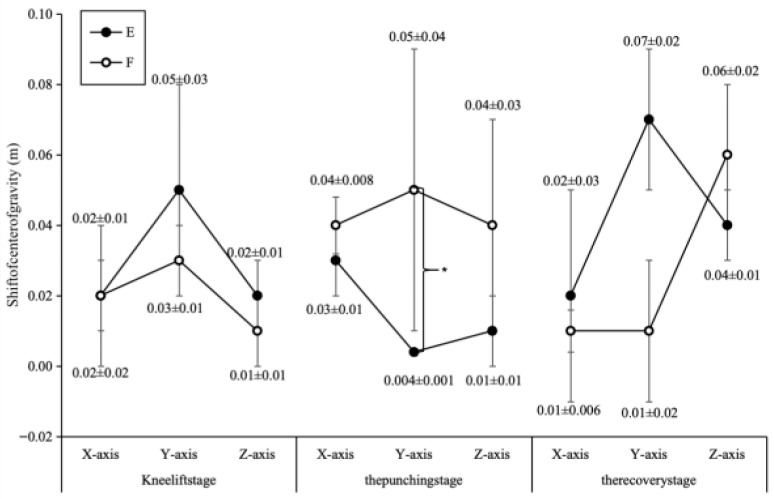
Comparison chart of center-of-mass displacement in technical movements between Group E and Group F.

During all three phases, both groups exhibited minimal COM displacement in the anterior–posterior (*X*-axis), lateral (*Y*-axis), and vertical (*Z*-axis) directions (centimeter-level; Figure 4). Displacement in all directions during the knee lift phase was highly similar between groups (all d < 0.2). However, during the striking phase, the elite group demonstrated significantly smaller vertical (*Y*-axis) COM displacement compared to the sub-elite group (E: 0.004 ± 0.001 m vs. F: 0.05 ± 0.004 m; t(18) = 3.45, *p* = 0.003, Cohen’s d = 1.84).

### 3.3. Joint Angles and Velocity

**Figure 5:**
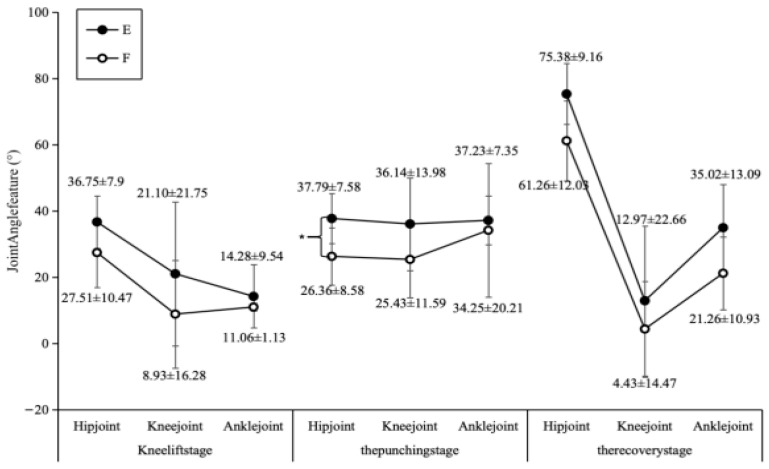
Comparison chart of joint angles in technical movements between Group E and Group F.

The elite group exhibited greater joint range of motion across most metrics (Figure 5). At the end of the recovery phase, the elite group’s hip joint angle (75.38 ± 9.16°) was significantly larger than that of the sub-elite group (61.26 ± 12.03°). Similarly, at the end of the knee lift phase, the elite group’s knee joint angle (21.10 ± 21.75°) exceeded that of the sub-elite group (8.93 ± 16.28°). During the striking phase (37.79 ± 7.58° vs. 26.36 ± 8.58°, d = 1.41) and recovery phase (75.38 ± 9.16° vs. 61.26 ± 12.03°, d = 1.33), the elite group’s hip joint angle was significantly greater than that of the sub-elite group’s.

**Figure 6:**
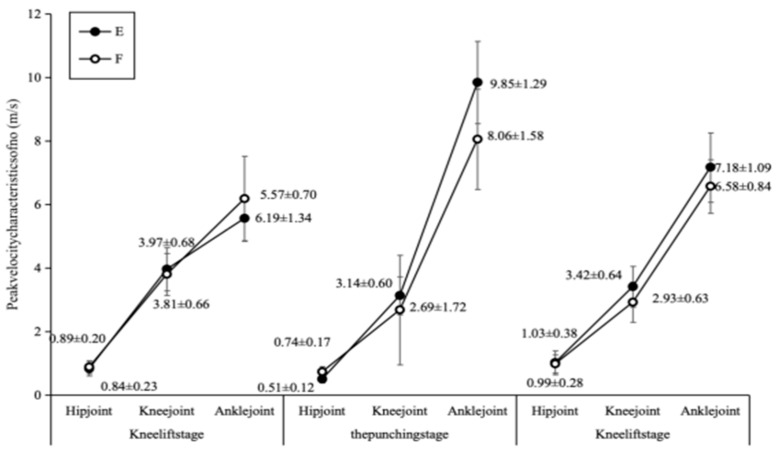
Comparison chart of joint angle and speed in technical movements between Group E and Group F.

Regarding angular velocities (Figure 6), the two groups exhibited distinct characteristics across joints and phases. During the striking phase, the elite group demonstrated a higher peak angular velocity at the ankle joint (9.85 ± 1.29 rad/s vs. 8.06 ± 1.58 rad/s). Conversely, during the knee lift phase, the sub-elite group exhibited a slightly higher peak angular velocity at the ankle joint (6.19 ± 1.34 rad/s vs. 5.57 ± 0.70 rad/s). Peak angular velocities at the hip and knee joints also showed phase-specific differences between the groups.

### 3.4. Surface Electromyography

#### 3.4.1. Knee-Raising Phase

**Figure 7:**
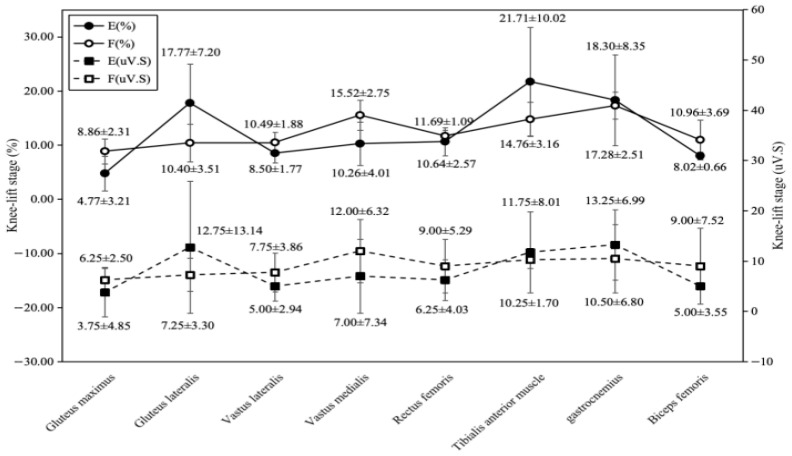
Comparison of comprehensive electromyography and muscle contribution between Group E and Group F during the knee-raising stage.

Distinct muscle activation patterns emerged between groups (Figure 7). The elite group exhibited the highest contribution percentage from the tensor fasciae latae (17.77 ± 7.20%), followed by the tibialis anterior (21.71 ± 10.02%). The sub-elite group’s highest contribution percentages were from the gastrocnemius (17.28 ± 2.51%) and vastus medialis (15.52 ± 2.75%). For IEMG values, the elite group showed greater activation in the gastrocnemius (13.25 ± 6.99 μV·s) and tibialis anterior (11.75 ± 8.01 μV·s), while the sub-elite group exhibited higher activation in the vastus medialis (12.00 ± 6.32 μV·s) and gastrocnemius (10.50 ± 6.80 μV·s).

#### 3.4.2. Striking Phase

**Figure 8:**
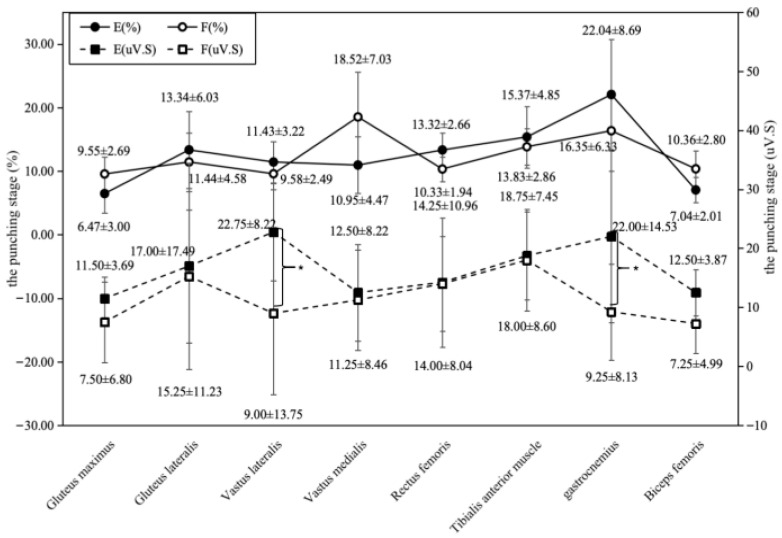
Comprehensive electromyogram and muscle contribution comparison chart for Group E and Group F during the punching stage.

Muscle activity intensity and contribution levels changed markedly during the core striking phase (Figure 8). In the elite group, the gastrocnemius showed the highest contribution percentage (22.04 ± 8.69%) and IEMG value (22.00 ± 14.53 μV·s). For the sub-elite group, the vastus medialis had the highest contribution percentage (18.52 ± 7.03%), and the tibialis anterior had the highest IEMG value (18.00 ± 8.60 μV·s). Additionally, the elite group’s vastus lateralis IEMG value (22.75 ± 8.22 μV·s) was significantly higher than that of the sub-elite group (9.00 ± 13.75 μV·s).

#### 3.4.3. Recovery Phase

**Figure 9:**
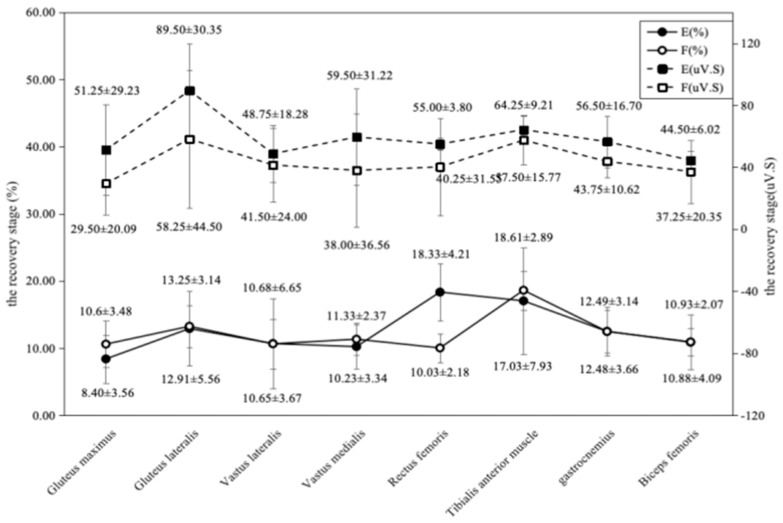
Comparison of comprehensive electromyography and muscle contribution between Group E and Group F during the recovery phase.

IEMG values of all muscles generally increased substantially during the recovery phase compared to the previous two phases (Figure 9). In the elite group, the tensor fasciae latae exhibited the highest IEMG value (89.50 ± 30.35 μV·s), followed by the tibialis anterior (64.25 ± 9.21 μV·s) and rectus femoris (55.00 ± 3.80 μV·s). The sub-elite group’s highest IEMG values were from the tensor fasciae latae (58.25 ± 44.50 μV·s) and tibialis anterior (57.50 ± 15.77 μV·s). In terms of contribution percentage, the rectus femoris stood out in the elite group (18.33 ± 4.21%), while the tibialis anterior was prominent in the sub-elite group (18.61 ± 2.89%), with a large effect size for this difference (d = 2.41).

Collectively, these data indicate that elite athletes differ systematically from sub-elite athletes in body control, joint range of motion, and muscle recruitment during the core power phase. Effect size analysis further highlights that differences are concentrated in the striking and recovery phases rather than the knee lift phase.

## 4. Discussion

This study systematically compared the biomechanical performance of elite versus sub-elite female karate practitioners during Mawashi Geri Jodan via integrated kinematic and sEMG analysis. Key findings confirmed our hypotheses, demonstrating that elite athletes appear to demonstrate quantifiable technical patterns that may reflect optimization for movement efficiency.

### 4.1. Kinematic Characteristics

#### 4.1.1. Technical Execution Efficiency

In combat sports, technical execution speed is a critical determinant of attack effectiveness. Faster movement completion reduces opponents’ reaction windows, directly influencing scoring success (Junior, 2015) [51]. This study found that elite athletes completed the full Mawashi Geri Jodan and core striking phase in significantly less time than sub-elite athletes, aligning with Kim et al.’s (2010) [16] report of higher kicking efficiency among elite taekwondo athletes. Shorter execution time may suggest neuromuscular system adaptation, although longitudinal studies are needed to confirm causality, indicating enhanced efficiency from movement planning to execution (Robalino et al., 2025) [24]. This not only demonstrates exceptional explosiveness and coordination but may also relate to optimized karate-specific knowledge structures developed through prolonged training. As Milazzo et al. (2016) [52] noted, enhanced perceptual and cognitive skills can improve decision-making accuracy and initiation speed. In high-level competitions, elite athletes seize scoring opportunities by shortening technical execution time, making this temporal advantage a crucial biomechanical hallmark of elite performance. Coaches should incorporate time constraints or rapid-response cues during routine training while maintaining movement standardization to simulate match tempo.

#### 4.1.2. Body Stability

Core stability is foundational to athletic performance (Kibler et al., 2006) [53]. During the striking phase, elite athletes exhibited significantly less vertical COM displacement than sub-elite athletes, indicating that maintaining trunk stability during force generation is critical to elite performance (Barbado et al., 2016) [54]. Non-essential body movement during striking results in power loss (Lenetsky et al., 2018) [55]. Elite athletes tended to maintain trunk stability, which may facilitate more direct force transfer from the supporting leg to the striking leg by maintaining trunk stability, emphasizing that training should prioritize core stabilizing control capacity under dynamic force generation rather than just absolute strength.

#### 4.1.3. Hip Joint Mobility

As the structural link between the axial skeleton and lower limbs, the hip joint transmits ground reaction forces upward and channels trunk/upper-body forces downward, serving as a pivotal node for power transmission (Zaghloul & Mohamed, 2018) [56]. The elite group demonstrated significantly greater hip joint mobility during the striking and recovery phases. In combat sports such as taekwondo and karate, effective roundhouse kicks are associated with rapid pelvic rotation and substantial hip mobility (Gavagan & Sayers, 2017) [57]. Greater hip joint range of motion (flexion, extension, external rotation) provides a longer acceleration pathway for the kicking leg (Dörge et al., 2002) [58]. Hip flexibility is consistently recognized as a critical factor in generating powerful kicks, facilitating optimal force generation and transmission through the kinetic chain without compensatory movements that compromise stability (Kellis, 2007) [59]. These findings further substantiate that maximizing hip joint range of motion is a pivotal biomechanical factor for enhancing kicking performance, underscoring the importance of hip mobility for achieving greater kicking range and height (Sørensen et al., 1996) [60].

### 4.2. sEMG Characteristics

#### 4.2.1. Muscle Synergy During the Striking Phase

Efficient kicking relies on synchronized, high-intensity recruitment of the lower limb extensor chain at impact (Sun et al., 2024) [61]. During the striking phase, elite athletes exhibited significantly higher IEMG values in the vastus lateralis and gastrocnemius compared to sub-elite athletes, indicating more complete and synchronous recruitment of primary prime movers in the lower limb power chain (Kibler et al., 2006) [53]. This aligns with the findings of Quinzi et al. (2013) [34] on enhanced neuromuscular control specificity in elite combat athletes, who exhibit higher muscle fiber conduction velocities and coactivation indices during specific movements, reflecting neural adaptation to prioritize fast-twitch motor unit recruitment. This heightened neural drive may contribute to greater striking force, though future studies incorporating kinetic measurements are warranted, and sub-elite athletes’ relatively dispersed and inadequate muscle recruitment represents a bottleneck in force development.

#### 4.2.2. Muscular Control During the Recovery Phase

Technical integrity encompasses rapid, proactive recovery to reposition the COM, defend against counterattacks, and prepare for consecutive strikes (Estevan, 2013) [62]. The elite group’s rectus femoris contribution rate during the recovery phase was nearly double that of the sub-elite group, a key finding. Neuromuscularly, the rectus femoris (a major hip flexor) is essential for powerful roundhouse kicks (Thibordee & Prasartwuth, 2014) [63], and its active recruitment for leg recovery is a critical component of elite technique (Hariri, 2018) [11]. We hypothesize that elite athletes’ robust rectus femoris activation during recovery reflects neuroadaptation enabling faster, more explosive concentric/eccentric contractions (Aagaard et al., 2002) [64], which directly influence rapid repositioning, balance maintenance, and preparation for subsequent attacks.

### 4.3. Limitations

Several limitations should be considered when interpreting these findings. First, the small sample size (N = 8) restricted statistical power and may limit generalizability (Bishop et al., 2021) [65]. As noted by Button et al. (2013) [66], small-sample studies are valuable for generating hypotheses but require cautious interpretation and replication in larger cohorts. While we observed large effect sizes (Cohen’s d > 0.8) for key variables, these findings should be considered preliminary until validated in larger, multi-center studies. Second, the cross-sectional design precludes establishing causality between observed differences and skill levels; longitudinal tracking is needed to elucidate the role of these biomechanical markers in long-term athlete development. Third, the controlled laboratory setting differs from actual competition in psychological load and dynamic opponent interactions. Fatigue significantly influences kinematic and sEMG parameters (Rodrigues et al., 2023) [67], while psychological factors (self-confidence and anxiety) may affect technical execution (Anastasiou et al., 2024) [68]. Future studies should validate findings in ecologically valid simulated competition settings, incorporating concurrent monitoring of physiological and psychological states.

Despite these limitations, this study objectively identified key indicators distinguishing karate athletes’ skill levels through comprehensive biomechanical analysis, thus deepening the understanding of Mawashi Geri Jodan biomechanics and providing empirical evidence for high-level training.

### 4.4. Practical Applications

By quantifying key kinematic and neuromuscular parameters, this study provides an objective basis for identifying individual technical deficiencies and comparing the characteristics of high-level athletes, thereby assisting coaches in designing targeted training interventions. Future research should expand the sample size and conduct longitudinal tracking to examine the evolution of biomechanical indicators over the course of training and their impact on long-term athletic performance. Additionally, it is recommended to extend the analytical framework to other fundamental karate techniques, such as gyaku tsuki and mae geri, to reveal common characteristics of elite performance and technique-specific determinants, thereby enhancing the robustness and generalizability of the research findings.

## 5. Conclusions

This study systematically compared the biomechanical characteristics of elite and sub-elite female karate athletes during the execution of Mawashi Geri Jodan using synchronized kinematic and surface electromyography (sEMG) data. The results showed that elite athletes completed the overall technique and the impact phase in a shorter duration, exhibited smaller vertical center-of-mass displacement during the impact phase, and demonstrated greater hip range of motion during both the impact and leg retraction phases, suggesting superior neuromuscular efficiency, trunk stability, and force transfer capability. Electromyographic analysis revealed that elite athletes displayed higher activation of the vastus lateralis and gastrocnemius during the impact phase and greater contribution of the rectus femoris during the leg retraction phase, reflecting optimized force production and leg retraction strategies. This study provides preliminary quantitative evidence for the specific training of elite karate athletes, although the findings warrant further validation with larger sample sizes.

## Figures and Tables

**Table 1:** Anthropometric characteristics of participants (mean ± SD).

	Elite Group (n = 4)	First Group (n = 4)	t	*p*
Height (cm)	170.0 ± 4.2	165.5 ± 2.4	−1.850	0.114
Weight (kg)	61.75 ± 3.5	56.0 ± 4.7	−1.965	0.097
Age (years)	22.7 ± 3.0	22.0 ± 1.1	−0.454	0.666

## Data Availability

The data supporting the findings of this study are included within the article.

## Abbreviations

The following abbreviations are used in this manuscript:

3D	Three-Dimensional
COM	Center of Mass
E	Elite Group
F	Sub-Elite Group
IEMG	Integrated Electromyography
sEMG	Surface Electromyography
SPFT	Special Physical Fitness Test
WKF	World Karate Federation
